# Reviewing the complexities of horseshoe kidney: insights into embryogenesis and surgical considerations

**DOI:** 10.25122/jml-2024-0325

**Published:** 2025-01

**Authors:** Ovidiu-Catalin Nechita, Daniel Badescu, Razvan-Ionut Popescu, Stefan Rascu, Razvan-Cosmin Petca, Justin Aurelian, Traian Constantin, Cristian-Valentin Toma, Viorel Jinga, Bogdan Geavlete

**Affiliations:** 1 Department of Urology, Carol Davila University of Medicine and Pharmacy, Bucharest, Romania; 2 Department of Urology, Prof. Dr. Theodor Burghele Clinical Hospital, Bucharest, Romania; 3 Medical Sciences Section, Academy of Romanian Scientists, Bucharest, Romania; 4 Department of Urology, Sf. Ioan Emergency Clinical Hospital, Bucharest, Romania

**Keywords:** Horseshoe kidney, congenital anomaly, renal fusion, renal vascular variations, kidney anatomical abnormalities, kidney embryogenesis

## Abstract

Horseshoe kidney (HSK) is a common renal malformation with unique and complex characteristics. A systematic literature search was conducted using PubMed and ScienceDirect databases. Several theories have been proposed regarding HSK formation, such as the close apposition of the kidneys during ascent through an arterial fork, lateral flexion of the trunk, and caudal embryonic rotation. Emerging evidence from animal models implicates notochord signaling and the sonic hedgehog pathway in HSK formation. The isthmus, a defining feature of HSK, is hypothesized to arise from ectopic mesenchymal tissue. The surgical anatomy of HSK is complex, given the variability in location, orientation, and blood supply. Both arterial and venous anatomy exhibit significant variability, raising questions about whether anomalous blood supply is a cause or a consequence of abnormal renal position. The isthmus usually contains functional renal parenchyma and fusion between the kidneys, primarily at the lower pole. While it is often stated that the inferior mesenteric artery is “held back” at the L3 level, this anatomical configuration is present in only 40% of cases. The review highlights the need for further research and provides a comprehensive overview of HSK knowledge.

## INTRODUCTION

Horseshoe kidney (HSK) is the most prevalent congenital renal fusion anomaly characterized by three major morphological abnormalities: ectopia, malrotation, and changes in vascular supply. The first recorded description of HSK dates back to 1522 when Jacopo Berengario da Carpi identified the anomaly during autopsy examinations [1]. HSK typically results from the fusion of the kidneys at their lower poles, forming an isthmus composed of either parenchymal or fibrous tissue [2].

The reported incidence of HSK varies in anatomical dissections, ranging from 0.15% to 0.48% [3]. While there is no confirmed genetic determination for HSK, it has been observed in identical twins and siblings within the same family [4]. HSK often remains asymptomatic in adults, but it can have significant implications for secondary renal pathologies such as urinary tract infections, hydronephrosis, and stone formation [5]. The detection of fusion anomalies occurs across different age groups, with HSK being diagnosed in small children as part of multiple malformations, in young adults during the diagnosis of delayed menarche associated with Turner syndrome, and incidentally in adults during routine radiological procedures for other reasons [6]. Despite numerous articles on renal fusion and ectopia, there is a lack of comprehensive reviews that systematically summarize the current understanding of HSK. Therefore, this study evaluated the existing literature on horseshoe kidneys' clinical anatomy, etiology, and embryology and summarized their surgical impact.

## MATERIAL AND METHODS

A comprehensive literature review was conducted using the ScienceDirect and PubMed databases to explore the topic of horseshoe kidneys. The search strategy utilized specific keywords such as 'horseshoe kidney', 'kidney fusion', and 'kidney vascular' to ensure a comprehensive coverage of relevant articles. A systematic approach was employed to identify suitable articles for the review.

**Figure 1:**
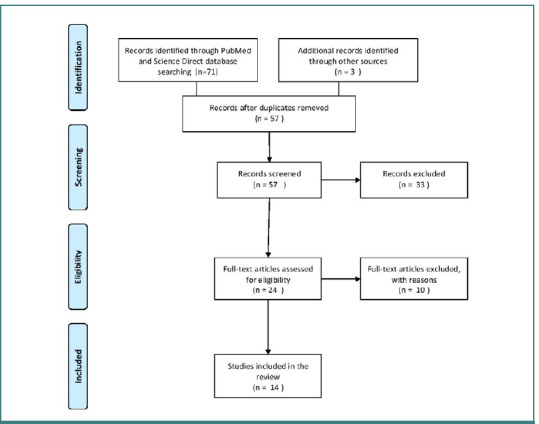
PRISMA flow diagram for the selection process

A total of 24 articles were selected for in-depth analysis from the initial search results. The search was limited to articles published in English without specific date restrictions, ensuring a broad range of information was considered. Following a thorough review and evaluation of the selected articles, 14 articles were deemed suitable for inclusion in the manuscript. Table 1 provides descriptions of the studies incorporated into the review, as well as their primary findings and associated limitations. These articles were carefully chosen based on their relevance, quality, and contribution to the understanding of HSK. The review included only studies pertinent to human surgical practices. Thus, articles based on animal models or in vitro experiments that did not directly apply to the surgical management of horseshoe kidneys in humans were excluded. Additionally, articles that may have had conflicts of interest, such as those authored or funded by pharmaceutical companies or medical device manufacturers, were excluded to avoid bias and maintain impartiality. The PRISMA flow diagram is shown in Figure 1.

**Table 1:** Key findings and research limitations identified across studies

No	Study details	Year	No. of patients	Sex	Measurement (Quantified)	Relevant Findings	Limitations
1	Humphries *et al.* [6]	2023	N/A	W, M	Frequency of abnormal results in fetuses with HSK compared to the general population.	Incidence: 1 in 500 people Higher incidence in males (2:1), associated with chromosomal disorders. Fusion at the inferior pole in over 90% of cases - Isthmus composition varies. Position often anterior to the aorta. Multiple theories on HSK development. Aberrant cell migration, altered signaling, genetic mutations.	Limited to the analysis of fetuses. Specific numbers not provided.
2	Murugapoopathy *et al.* [10]	2020	N/A	N/A	Prevalence of CAKUT	Estimated at 4–60 per 10,000 births: variability due to sample size, diagnosis method, and ethnic differences.	Limited information on mild CAKUT cases. No systematic noninvasive method to assess nephron number.
3	Pollak & Friedman [13]	2020	N/A	N/A	Genes implicated in kidney diseases. Types and classifications of genetic variations.	Description of single-nucleotide polymorphisms (SNPs), insertions/deletions, structural variations, and their effects. Examples of genes influenced by evolutionary pressures and their dual effects on health, such as APOL1 and UMOD.	Variability in the impact of genetic variations. Different evolutionary pressures affecting gene behavior. Species-specific differences in gene behavior.
4	Srinivas *et al.* [21]	2016	N/A	N/A	Various imaging modalities, including ultrasonography, multidetector computed tomographic urography, magnetic resonance imaging, and intravenous urography, were utilized to evaluate congenital kidney and urinary tract anomalies.	Various congenital kidney and urinary tract anomalies were discussed, including renal agenesis, Herlyn-Werner-Wunderlich syndrome, Zinner’s syndrome, supernumerary kidney, horseshoe kidney, pancake kidney, and ectopic kidney.	Excludes clinical management and outcomes, offering only an imaging-centric perspective
5	Natsis *et al.* [17]	2014	250	W, M	Morphological appearance, Associated anomalies, and diseases	HSK occurs in approximately 1 of 400-600 individuals, with a higher frequency in men (2:1 ratio). Prenatal diagnosis is possible in the first trimester using high-frequency transvaginal sonography. Associated severe anomalies or malformations found in 23% of patients, with a higher frequency in children than adults.	Limited to cadaveric studies. Gender distribution not specified for cadavers.
6	Ichikawa *et al.* [30]	2011	105	W,M	The frequency and diversity of abnormal IVC occurrences in individuals with horseshoe kidneys detected through multidetector row computed tomography (MDCT).	Anomalous inferior vena cava (IVC) found in 28.6% of patients with horseshoe kidneys. Variations of IVC identified in 5.7% of patients, including pre-isthmic IVC with retrocaval ureter, double IVCs posterior to the horseshoe kidney, left IVCs posterior to the horseshoe kidney, and azygos continuation of the IVC.	The study is limited by its retrospective design and may not capture all IVC anomaly cases in horseshoe kidney patients.
7	Tripathi *et al.* [16]	2010	N/A	N/A	Study of embryonic kidney development on mice embryos.	Metanephric kidney development is influenced by axial structures such as the notochord and floor plate. Ablation of the notochord and floor plate led to kidney fusion but not renal agenesis.	The study is conducted on mice embryos. The relevance to human kidney development needs further investigation.
8	Glodny *et al.* [2]	2009	209	W, M	Morphological appearance	High variability in the vasculature of HKs and CFEs that could not be classified.	Specific details on patient sex not provided
9	Fazio *et al.* [26]	2003	3	W, M	CT, angiography, surgical margin (1 cm).	Two patients had renal cell carcinoma (RCC), and one had transitional carcinoma (TCC). Surgical approaches discussed. Survival exhibited a correlation with the grade and stage of the disease.	A limited number of cases.
10	Yoshinaga *et al.* [18]	2002	814	W, M	Kidney dimensions (length, width, thickness), ureter diameter, arterial and venous anatomy.	The segmented arterial supply pattern is similar to that of a normal kidney.	Single-case study. Limited sample size, age of the patient, lack of embryological explanation.
11	Doménech-Mateu&Gonzalez-Compta [24]	1988	1	Embryos	The embryogenesis of HSK in a human embryo measuring 16 mm in crown-rump (CR) length.	Proposed new theory on embryogenesis of horseshoe kidney malformation. Suggested origin from posterior nephrogenic area of the epiblast.	Limited to a single embryo specimen.
12	Castro & Green [22]	1975	1	W	Hydronephrosis, renal calculi, urinary infection, renal pelvis tumor	Occurrence of tumors in horseshoe kidney is unusual. 20 cases with carcinoma of the renal pelvis previously described.	Limited to a singular case study
13	Friedland & de Vries [27]	1975	1	Embryos	Measured sagittal and transverse distances, including umbilical artery separation, for anatomical analysis.	Renal ascent was observed relative to the umbilical arteries, tail fold, and vertebral column. The upper pole of the renal blastema displayed a comet-like extension. Furthermore, inhibiting growth during specific developmental stages led to renal ectopia and fusion.	Some sections lacked specific quantified measurements. Findings are based on embryonic development and may not directly apply to postnatal renal anatomy.
14	Kilpatrick [23]	1967	40	W, M	Various complications, including hydronephrosis, infection, stones	Most common finding is unilateral or bilateral hydronephrosis with associated complications. Some patients had recurrent infections, stones, or tumors in the renal pelvis. Surgical treatments included symphysiotomy, nephropexy, and nephrectomy. Results were mixed, with some cases improving while others needed further interventions.	No long-term follow-up data were available for evaluation.

W, women; M, men; E, embryos; HSK, horseshoe kidney; CAKUT, Congenital Anomalies of the Kidney and Urinary Tract; APOL1, Apolipoprotein L1; UMOD, Uromodulin; CT, Computed Tomography; CFE, Crossed fused ectopia; SNPs, Single-Nucleotide Polymorphisms; MDCT, Multidetector Row Computed Tomography; RCC, Renal Cell Carcinoma; TCC, Transitional Carcinoma

## RESULTS

Table 1 displays the characteristics and limitations of each study included in this review.

### Embryogenesis

During the intricate process of embryogenesis, each kidney undergoes development involving two distinct cell populations: the ureteric bud and the metanephric blastema [7]. The ureteric bud gives rise to the collecting system, while the metanephric blastema contributes to the formation of the functional kidney. These two structures converge in the upper sacral region (specifically, S1-S2) through reciprocal induction during the fourth week of development [8]. This pivotal process is essential for the proper establishment of the urinary system. Disruptions in this delicate course can give rise to a diverse array of congenital urological conditions [9].

Reference textbooks indicate renal fusion anomalies typically manifest between the fourth and sixth weeks of embryonic development [10]. However, certain authors propose an extended timeframe of up to nine weeks, particularly in cases involving a fibrous isthmus [11]. The etiology of horseshoe kidneys encompasses multiple hypotheses, as they represent the outcome of various underlying factors. These potential mechanisms include positional factors and anomalous fusion arising from proximity, abnormalities in the migration of metanephric cells, influences from the intrauterine environment such as maternal factors and exposure to teratogens, as well as genetic and chromosomal aberrations [12].

### Genetic associations in HSK

The genetic etiology of HSK in humans remains unclear; however, several regulatory processes involved in kidney development have yet to be fully understood, offering potential insights into its underlying causes. Animal models have implicated the role of the notochord in determining the positioning of metanephric tissue. Additionally, the depletion of Sonic Hedgehog (SHH), an axial signaling molecule, has been demonstrated to be sufficient in inducing renal fusion, even in the presence of the notochord [13].

There is a well-documented male predominance with a male-to-female ratio 2:1 [14]. Familial clustering has been observed in some instances, including father-son pairs and monozygotic twins. Three siblings exhibited horseshoe kidneys in one family, while the mother had a malrotated kidney [15]. These instances provide circumstantial evidence supporting the potential contribution of fetal genetic programming to the etiology of the horseshoe kidney. However, there are also reports of monozygotic twins where only one twin is affected, suggesting additional factors at play [16].

Some researchers propose that urological malformations associated with chromosomal abnormalities may arise, in part, due to delayed development of the nephrogenic blastema and the ureteric bud [17]. HSK is observed in approximately two-thirds of individuals with Edwards syndrome, while the incidence of Down syndrome is likely less than 1% [18]. In Turner syndrome, horseshoe kidneys occur in 14–20% of patients, with a lower occurrence in mosaicism patients [19]. These findings highlight the associations between chromosomal abnormalities and renal malformations in different genetic conditions [20].

### Causative factors and anatomical considerations in fusion defects of the kidney

Abnormal fluctuations in fetal growth and ventral flexion within the confined true pelvis have been implicated as potential causes of fusion defects [21]. It has been observed that normal embryos have closely positioned metanephric blastemas prior to renal ascent [22]. The prevailing view suggests that the renal masses come into close apposition during ascent as they pass through an arterial fork. However, there is inconsistency regarding the specific anatomical entity at which this occurs, with some sources indicating the aortic bifurcation while others point to the umbilical arteries [23]. Generally, a higher degree of fusion corresponds to a more ectopic renal position [24].

Renal fusion can also be triggered by flexion or rotation of the caudal end or spine during renal ascent, even within the normal range of developmental variability [25]. Similarly, slight alterations in the position of key arteries, such as the umbilical or common iliac arteries, can influence the path of renal migration and potentially lead to fusion [26]. Additionally, it is worth noting that even in individuals without fusion anomalies, both kidneys share a common perirenal space that crosses the midline [27].

The occurrence of fusion anomalies in both symmetrical and asymmetrical patterns provides further insights into their etiology [28]. Symmetrical HSKs are believed to result from factors that affect both renal masses equally, such as abnormal growth or ventral flexion within a constricted embryonic pelvis [29]. Delayed straightening of the caudal fetus may also contribute to renal ascent delay and subsequent fusion. On the other hand, asymmetrical or laterally fused HSKs result from differential displacement of the renal masses [30]. Factors such as lateral flexion of the trunk or rotation of the caudal embryo have been proposed as potential causes, and the association of asymmetrical HSKs with vertebral conditions supports this hypothesis [31]. Considering that the genitourinary system and vertebral column originate from distinct components of the mesoderm but develop synchronously, this association may also reflect a developmental field defect or sequence.

Aberrant cell migration during embryogenesis potentially contributes to the varied composition of the isthmus in HSK, whether it predominantly consists of renal parenchyma or fibrous tissue [32]. Studies have reported that the isthmus comprises renal parenchyma in approximately 80% to 85% of cases[33]. Mutations leading to abnormal renal hyperplasia and dysplasia in the medial portion of the metanephros may reduce the distance between the two metanephros, influencing renal cell migration [34].

The bridge that connects the two renal masses in HSK exhibits variable anatomical relations and substance [35]. Its midline or lateral position to the vertebral column determines whether the HSK is symmetric or asymmetric [36]. Asymmetrical configurations are more commonly left dominant [37]. In approximately 80% of cases, the isthmus contains functional renal parenchyma, presenting a challenge for surgeons. Fusion between the kidneys primarily occurs at the lower pole in over 90% of cases, but upper pole fusion, resulting in an 'inverted horseshoe', and fusion at both poles, leading to a 'disc kidney', are also observed [38]. Additionally, double ureters may be present on one or both sides. In rare instances, one or both ureters may pass posterior to the isthmus [39].

The typical course of the isthmus involves crossing anterior to the great vessels, although posterior crossing or running between them occurs rarely [40]. Understanding the anatomical variations and substance of the isthmus in HSK is crucial for surgical planning and interventions, ensuring optimal patient outcomes.

In rarer instances where the isthmus primarily consists of fibrous tissue rather than renal parenchyma, mechanical fusion could be attributed to caudal embryo rotation or lateral trunk flexion [41]. Moreover, patients with HSK variants are three times more likely to have concomitant congenital vertebral anomalies, highlighting the association between axial skeleton growth abnormalities and HSK [42].

Considering the impact of altered growth rates in various organs, including the kidney, abnormalities associated with the axial skeleton's development may lead to a lack of appropriate skeletal support [43]. This can increase the risk of compression, affecting the normal functioning of organs [44]. Understanding these associated conditions and potential etiologies is crucial for comprehensive management and appropriate monitoring of patients with HSK, ensuring timely interventions and reducing the risk of complications [45].

### Vascular variations in horseshoe kidney

During the kidney's developmental process, various sources of vascular supply are gained and lost as it ascends. The pelvic region receives vascular supply from the median sacral artery and internal and external iliac vessels [46]. Subsequently, the kidney may receive direct supply from the aorta or branches, such as the common and inferior mesenteric arteries, before the renal artery is gained [47]. As cranial migration progresses, the caudal arteries degrade, and reports indicate that persistent embryonic arteries can inhibit cranial migration [48]. The relationship between anomalous blood vessels and abnormal renal position raises the question of whether these vessels are the cause or result of the abnormality. This is particularly relevant in cases of inverted HSK and when there are variable relations with the great vessels [49]. Understanding the vascular anatomy of kidney fusion anomalies is crucial for comprehensive evaluation and management.

The origin, number, and size of renal vessels in kidney fusion anomalies display significant variation based on the termination point of the kidney's ascent during development. The proposed vascular pattern of the HSK, introduced by Graves in 1969, holds historical significance but has been further elucidated with the advancement of imaging techniques [15]. Detailed analysis of the vascularization of horseshoe kidneys has revealed that renal arteries can originate from the abdominal aorta, common iliac arteries, and the inferior mesenteric artery [50,51].

Recent studies utilizing advanced imaging techniques have shed light on the extensive variations in vascular anatomy within HSK [52]. These investigations have revealed that, on average, each renal unit in a study of 90 HSKs is supplied by two or more feeding arteries, with a higher number of arteries observed on the right side [53]. The cranial renal arteries exhibited greater consistency in position and tended to originate more posteriorly from the aorta than the caudal arteries.

The percentage of vessels originating from the aorta decreased progressively with the number of arising arteries [54]. Despite the anomalous nature of these vessels, they maintain the segmental pattern characteristic of normal renal vasculature. Attempting to ligate or divide these vessels may result in ischemic necrosis due to their autonomous blood supply. Of particular interest is the blood supply of the isthmus, which displays significant variation and can potentially supply the entire kidney in some cases [55]. The isthmus receives its blood supply from various sources, including the main renal artery, branches from the abdominal aorta above or below the isthmus, the common iliac artery, or the inferior mesenteric artery [56].

Another retrospective cohort study emphasized the complexity of arterial origins in kidney fusion anomalies. Among more than 200 cases analyzed, only a small percentage featured a simple situation with one artery on each side. The study highlighted a wide range of potential arterial origins, including the common iliac artery, median sacral artery, internal iliac artery, external iliac artery, iliolumbar artery, and phrenic artery. Additionally, the study considered cases where arteries supplied portions of the contralateral kidney, which occurs in approximately 25% of horseshoe kidney cases [57]. Overall, advanced imaging techniques and comprehensive studies have provided valuable insights into the vascular anatomy and blood supply patterns within HSKs, contributing to a deeper understanding of this intriguing renal anomaly.

High incidences of kidney vein anomalies are frequently observed in HSKs, with a reported rate of 23% [58]. These anomalies can involve inferior vena cava (IVC) abnormalities such as double IVC, left IVC, and pre-isthmic IVC. The prevalence of IVC variations in HSKs is approximately ten times higher than in the general population (5.7%). Double IVC or pre-isthmic IVC cases have been documented in case study reports [59]. Additionally, HSKs can be associated with double superior vena cava, which often co-occurs with cardiovascular malformations [60].

General conclusions regarding the vasculature of HSKs have been derived from a retrospective cohort study conducted by Davidovic *et al*. [61]. The cranial vessels of the kidneys on both sides typically exhibit consistent patterns, with the second artery on the left side positioned precaval and the second vein on the left side typically retro-aortic. However, the caudal kidney vessels tend to be more ventrally located and display greater variation [62]. These significant variations in vascular supply pose challenges for upper tract surgery, kidney transplantation, and surgical and endovascular procedures. Understanding the intricate vascular variations within HSKs is crucial for surgical planning and avoiding potential complications in various urological and vascular interventions.

### Upper urinary tract variations in HSK

The upper urinary tracts of HSKs exhibit significant variations in terms of their number and origin. Usually, the calyces are situated in the upper two-thirds of each kidney, although the isthmus may be drained by an external calyx or an independent ureter. Anatomical variations of the urinary tracts associated with HSK have been reported in case studies [63].

For instance, Shen *et al*. [19] described a case of HSK with retrocaval ureters, while Afzal *et al*. [20] presented bilateral ureteral duplication. Changes in the position of the ureter within HSKs, where they typically terminate in the bladder but can also exhibit ectopic positions, can directly result in secondary hydronephrosis and uretero-pelvis junction (UPJ) obstruction. UPJ obstruction often arises due to the high insertion of the ureters into the renal pelvis, causing delayed pelvic emptying [64].

Rotation abnormalities of the kidneys during gestation, particularly during the 6th to 8th week, can lead to ureteral obstruction in HSKs, where malrotation is a common anatomical abnormality characterized by incomplete or non-rotations [65]. In HSKs, deviations from the normal kidney hilum rotation, such as incomplete rotations or non-rotations, are frequently observed, highlighting their significant role in ureteral obstruction, while hyper-rotation or reverse rotation can also occur [66].

The clinical implications of upper urinary tract abnormalities in HSKs encompass a variety of consequences. These include the prevalence of nephrolithiasis in 16% to 60% of HSK cases and the susceptibility to urinary tract infections [67]. These complications highlight the significance of addressing and managing upper urinary tract abnormalities in individuals with HSKs to mitigate potential health complications. Understanding the changes and variations in the upper urinary tract of HSKs is essential for effectively managing and treating associated complications, such as hydronephrosis, UPJ obstruction, and urinary stone formation. Proper diagnosis and comprehensive evaluation of these abnormalities are crucial in providing appropriate care for individuals with HSKs [68].

### Clinical consequences of upper urinary tract abnormalities in HSKs

HSK, characterized by its unique anatomical position in the lower part of the abdomen and the presence of the isthmus across the midline, poses an increased risk of blunt abdominal trauma [69]. In cases of lower abdominal trauma among individuals with HSK, especially if hematuria is present, prompt radiological investigation becomes crucial to evaluate potential hematoma formation and associated injuries [70].

HSK is typically asymptomatic and is often incidentally discovered. However, when symptoms do arise, they are usually associated with obstruction, stones, or infection [71]. UPJ obstruction, occurring in approximately 35% of patients with HSK, is the most prevalent finding and can be attributed to factors such as a high insertion of ureters into the kidney pelvis and the crossing of the ureter over the HSK isthmus [72]. UPJ obstruction in HSK is diagnosed using imaging techniques like CT urography or intravenous pyelography, which show characteristic features like a large pelvis with a high-riding ureter [73]. Treatment options include laparoscopic pyeloureteroplasty, while the division of the isthmus, a historical technique, poses risks of complications.

Nephrolithiasis, the formation of kidney stones, is prevalent in HSK. It is commonly observed, with a prevalence of 16% to 60% [74]. Kidney stones in adults with HSK are significantly higher than in the general adult population [75]. Several factors contribute to the development of kidney stones in HSK patients, including atypical positioning of the ureter in the renal pelvis, high insertion of the ureter at the UPJ, metabolic abnormalities such as hypercalciuria, hyperoxaluria, hyperuricosuria, and hypocitraturia commonly found in HSK patients, and the coexistence of HSK with medullary sponge kidney, which further elevates the risk of stone formation [76]. Calcium-based stones are the most prevalent type, accounting for approximately 89.2% of kidney stones in patients with HSK. Other types of stones include struvite stones (4.2%), uric acid stones (3.8%), and stones with other causes (2.8%) [77]. The risk of developing large staghorn stones in HSK patients is higher [78]. Treatment options for kidney stones in HSK are similar to those used for individuals with normal kidneys and may include extracorporeal shock wave lithotripsy (ESWL) or retrograde intrarenal surgery (RIRS) [79]. However, percutaneous nephrolithotripsy (PCNL), commonly used for staghorn stones, can be challenging in patients with HSK due to the unique orientation of the kidney calyx [80]. Research by Eryildirim *et al*. suggests that PCNL and RIRS are safe and effective minimally invasive procedures for stone removal in HSK patients [81].

In addition to urinary tract complications, HSK poses an increased risk of urinary tract infections (UTIs), particularly ascending infections, the most prevalent type commonly attributed to vesicoureteral reflux in HSK patients. The combination of reflux disease, stasis, and stone formation in HSK predisposes patients to UTIs, which can occur in up to one-third of HSK cases [82].

Furthermore, HSK is recognized for its association with a diverse range of benign and malignant tumors. The heightened susceptibility to malignancy in HSK is believed to stem from teratogenic factors present at birth [83]. Among HSK tumors, renal cell carcinoma is the most prevalent, constituting approximately 45–50% of cases [84]. Transitional cell carcinoma, linked to a fourfold relative increased risk in HSK patients, represents around 20% of tumors and is correlated with chronic infection, stone formation, and obstruction in the upper urinary tract. Additionally, HSK patients have a higher incidence of carcinoid and Wilms tumors compared to the general population [85]. The development of renal carcinoid tumors is approximately 60 times more likely in individuals with HSK, while Wilms tumor accounts for around 30% of malignant tumors in this patient population [86].

Surgical treatment of tumors in HSK is challenging due to the fused kidney's unique anatomy, requiring specialized urological centers for minimally invasive approaches like laparoscopic and robot-assisted techniques. Partial nephrectomy, aimed at preserving kidney function, is an option for cortical tumors in HSK despite the higher risk of complications.

## DISCUSSION

### Advances in surgical management of HSK: embracing minimally invasive techniques and precision planning

The intricate vascular variations and upper urinary tract abnormalities observed in HSK have significant clinical implications. These anomalies pose challenges in terms of diagnosis, surgical planning, and management of complications. Nephrolithiasis, urinary tract infections, and obstructive conditions such as UPJ obstruction are common complications in HSK patients. Effective management strategies, including minimally invasive and specialized surgical techniques, are vital in optimizing patient outcomes [87].

HSK patients have an increased susceptibility to various benign and malignant tumors, notably renal cell carcinoma, transitional cell carcinoma, carcinoid, and Wilms tumor. This heightened risk underscores the importance of vigilant monitoring and early intervention in this patient population [88].

The coexistence of upper urinary tract abnormalities, vascular variations, and tumor predisposition in HSK necessitates a comprehensive evaluation approach. Early detection, accurate diagnosis, and tailored management are pivotal in mitigating the health risks associated with this congenital anomaly [89].

The surgical management of HSK, a rare congenital anomaly, presents unique challenges due to its complex anatomy. Strategies for partial nephrectomy in these cases have significantly evolved, focusing on minimally invasive techniques such as laparoscopic and robot-assisted surgery [90]. These modern approaches are distinguished by their effectiveness and reduced invasiveness, providing promising results in terms of renal function preservation and effective tumor management [91].

One of the most notable advancements in this field is the use of indocyanine green fluorescence guidance, as described in the study of Imai *et al*. [31]. This technique significantly improves tumor visualization during surgery, crucial in complex anatomies like HSK. The fluorescence provides a clear demarcation between tumorous and normal tissue, facilitating the preservation of healthy renal tissue.

Another promising technique is robot-assisted partial nephrectomy, which offers greater precision and flexibility. One study [92] highlights the advantages of robot-assisted partial nephrectomy for renal cell carcinoma in the isthmus of HSK, emphasizing its enhanced visualization and surgical precision, decreasing post-surgical complications, and better preservation of renal function.

Additionally, preoperative and intraoperative planning plays a crucial role in the success of these surgeries. The use of hyper-accurate three-dimensional virtual models, as presented in the study by Campi *et al*. [27], has proven to be a valuable tool. These models allow surgeons to plan and simulate the surgery in advance, reducing surgical time and improving outcomes.

However, it is also important to mention that open surgery remains a viable option in some cases. The study done by Romeo *et al*. [34] illustrates that, although minimally invasive techniques are preferable, open surgery may be necessary in certain situations, especially when tumor masses are large or in challenging locations.

In summary, the surgical management of HSKs has advanced considerably, focusing on preserving renal function and minimizing morbidity. The choice of surgical approach should be based on the patient's specific anatomy, tumor characteristics, and the surgeon's experience. As surgical technologies and techniques continue to evolve, we are likely to see even greater improvements in outcomes for patients with this complex condition.

In the future, research should prioritize deepening the genetic factors underlying HSK, providing more detailed insights into vascular variations, and enhancing the precision of surgical procedures. These advancements will play a pivotal role in elevating the quality of clinical decision-making and patient management, particularly concerning complications and malignancies associated with HSK.

## CONCLUSION

The etiology of the horseshoe kidney involves a combination of genetic factors, abnormal embryogenesis, and environmental influences. It is associated with diverse anatomical and vascular variations, including fusion defects, vascular anomalies, and upper urinary tract variations. Therefore, a comprehensive understanding of the complex anatomy, embryogenesis, genetic associations, and vascular variations of horseshoe kidney is crucial for accurate diagnosis, surgical planning, and effective management of patients with this condition.

## Data Availability

All information included in this review is documented by relevant references.
